# Insecticide susceptibility status of Anopheles stephensi against novel insecticides in Eastern Ethiopia

**DOI:** 10.21203/rs.3.rs-5511709/v1

**Published:** 2024-12-02

**Authors:** Ephrem Abiy, Teshome Degefa, Meshesha Balkew, Hailu Merga, Eshetu Alemayehu, Anteneh Mitiku, Ming-Chieh Lee, Guyin Yan, Delenasaw Yewhalaw

**Affiliations:** PMI Evolve Project Abt Global, Addis Ababa, Ethiopia; School of Medical Laboratory Sciences, Institute of Health, Jimma University, Jimma, Ethiopia; PMI Evolve Project Abt Global, Addis Ababa, Ethiopia; Department of Epidemiology, Institute of Health, Jimma University, Jimma, Ethiopia; Department of Epidemiology, Institute of Health, Jimma University, Jimma, Ethiopia; PMI Evolve Project Abt Global, Addis Ababa, Ethiopia; Program of Public Health, University of California at Irvine, USA; Program of Public Health, University of California at Irvine, USA; School of Medical Laboratory Sciences, Institute of Health, Jimma University, Jimma, Ethiopia

## Abstract

**Background:**

Anoph*eles stephensiwas* known to be local malaria vector in South East Asia but recently found expanding to the horn of Africa including urban areas of Ethiopia. Recent studies indicated that *An. stephensihave* high level of insecticide resistance to pyrethroid (Deltamethrin, permethrin and alpha-cypermethrin), Carbamates (Bendiocarb and Propoxur) and organophosphates (pirimiphos-methyl). The aim, of this study was to evaluate the susceptibility of *An. stephensi* from Diredawa against broflanilide, chlorfenapyr, clothianidin and pyriproxyfen.

**Methods:**

A standard diagnostic doses of broflanilide, chlorfenapyr, clothianidin and pyriproxyfen were tested, using the revised WHO bottle bioassay test protocol, against wild adult *An. stephensieared* from larval collections from urban artificial larval habitats in Dire Dawa.

**Results:**

The 60 minutes knock down result indicated 85%, 76% and 14% against brofilanilide, clothianidin and clorfenapyr respectively. No KD was observed in controls and PPF. Complete mortality of *An.stephensiat* 24 hours was observed against broflanilide (9ug/bottle) and clothiandin (10ug/bottle). Also 66% mortality at 24 h and 100% mortality at 48 h post exposure against clorfenapyr (100ug/bottle) were observed. In case of PPF (100ug/bottle), no mortality at 24 h, 11% at 48 h. and 15% at 72 h were recorded and no mortality in all controls.

In addition, 100% of oviposition were observed in controls and no oviposition were observed in PPF exposed mosquitoes. These results suggest that all three types **of insecticides namely: Broflanilide, Chlorfenapyr** and Clothianidin showed higher mortality than pyriproxyfen.

**Conclusion:**

Full susceptibility to the three novel insecticides were observed and full oviposition inhibition were observed in wild reared larvae *An. stephensi* exposed to PPF. Therefore, this study recommends using these novel insecticides to control *An. stephensi* in Ethiopia.

## Background

Malaria is a diseases caused by a protozoal parasite *Plasmodium* and there are four distinct species infected humans: *P falciparum, P vivax, P ovale* and *P malariae* [1, 2]. The species differ in regards to their morphology, details of their life cycles, and their clinical manifestations and severity. Recently *P knowlesiis* known to cause fatal malaria in Malaysia, South east Asia and is a threat to the globe [1–3].

Globally, 249 million malaria cases and 608,000 deaths were reported and 95% of the cases were from 29 high burdened countries. The four countries (Nigeria, Democratic Republic of Congo, Uganda and Mozambique) from Africa share half of the global malaria case [2].

The 2022 WHO Global report also indicated an increment of 5 million malaria cases than the 2021 and this is because some endemic countries experienced an increased case among which Ethiopia shared more than 1.3 million cases [2].

Climate change, war and conflicts, biological threats like parasite gene deletion and drug resistance and vector insecticide resistance exacerbates malaria transmission and changes the diseases dynamics affecting efficacy of diagnostic and control interventions.

In addition, the invasion and expansion of exotic *An. stephensito* the horn of Africa might increase the challenge of control and elimination of malaria [4, 5]. Its expansion is estimated to pose a significance change on the epidemiology of malaria and perhaps will impose severe transmission of malaria in Africa[5]

For the first time, the presence of *An. stephensi* in Ethiopia was reported in 2016 in Jigjiga area, Somali region by Carter [6] and a study conducted by PMI Ethiopia further indicated it’s geographical expansion to Afar, Oromia and Amhara regions [7, 8]. More recently, a study showed It’s expansion to the Western and Southern part of the country, BenishangulGumuz Region, Sidama and Arbaminch [9–11].

Different studies indicated that *An. stephensi* have high level of insecticide resistance against insecticides that has been in use like pyrethroid (Deltamethrin, permethrin and alpha-cypermethrin), Carbamates (Bendiocarb and Propoxur) and organophosphates (pirimiphos-methyl) and organochlorates in Arabian Peninsula and Asia but In Horn of Africa some studies indicated resistance to only Pyrethroid, Organophosphate and Carbamates [2, 8, 12–14].

There is a need for adaptation of new novel insecticides and effective vector control tools as part of Insecticide resistance management. As a result, the study aimed to evaluate the susceptibility status of *An. stephensi* against four novel insecticides: Cholrfenapyr, clothianidin, Broflanilide and pyriproxyfen in Eastern Ethiopia. Moreover, this study also evaluated the sterilizing effect of pyriproxyfen against *An. stephensiwith* WHO bottle bioassay in Eastern Ethiopia.

## Methods

### Study setting and design

**Figure 1:**
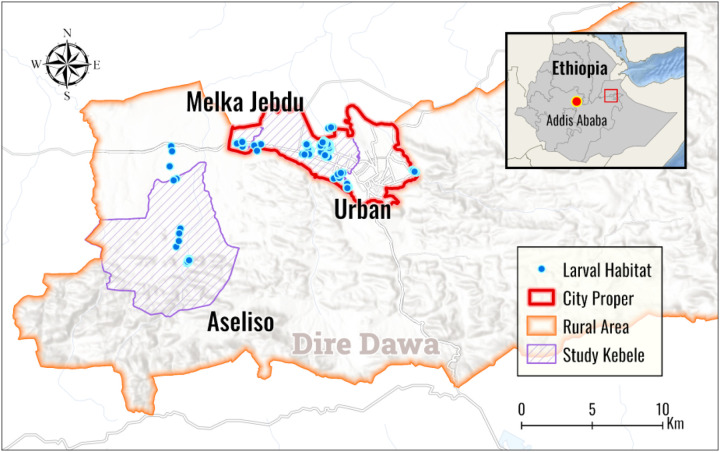
Map of the study area (Dire Dawa, 2024)

Experimental study was conducted in Dire Dawa City Administration located in the Longitude of 9.615240^0^, Latitude 41.8360^0^, at an altitude of 1174m and located 515km East of Addis Ababa (Fig. 1).

Rapidly ongoing urbanization, manufacturing, marketing, growing and continued trade, import and export through longtime railway are among features of Diredawa. It is an arid area where people store water for long time in an artificial containers and bricks. Expanding urbanization involves construction of modern houses with bricks and as a result there are evenly distributed brick making factories that use Uncovered water tankers, this helps the vector to easily breed and expand [7, 15, 16]. Moreover, high level of trade exchange and population movement occurs and act as an entry border to Ethiopia via Djbouti and this population movement might be linked it with *An. stephensiinvasion* to Ethiopia [7].

Annual report malaria cases report in the study area indicated 1,671 malaria cases at which 62.3% *P falciparum*, 36.3% *P vivaxand* 1.4% were Mixed cases. Malaria cases were peak in May- June, 2023.

### Test insecticides

Four classes of insecticides were used to conduct the insecticide susceptibility status of *An.stephensifrom* Diredawa. These insecticides are:

Chlorfenapyr (pyrrol) group insecticide and functions as an uncoupler of the oxidative phosphorylation which that kills the exposed mosquitoes through locking the ATP(Adenosine triphosphate) power house in the mithochondoria [17, 18].

Clothianidin (neo-nicotiniod) affects the mosquitoes through arresting the receptor *Acetaylcholine* channel of the nervous system [19].

Broflanilidae (meta diamide) class of insecticide with noncompetitive antagonist (NCA) action against GABA (gamma-butyric amino acid), NCAs inhibit permeation of chloride ion and induce hyper excitation and convulsion and kills the mosquito. BRF is also known by its trade name VectronT500 [20, 21].

Pyriproxyfen (Juvenile hormone analogue), are not insecticides nor intended to directly kill rather Juvenile hormone mimics potentially be used in vector control and acts on the mosquito embryogenesis, morphogenesis, reproduction generally aiming on inhibiting/reducing the fertility and fecundity of adult female mosquitoes; having an opportunity to a reduction in the density of the next generation of vectors (offspring) [22–27]. All the test insecticides (AI) and solvents (Mero & Acetone) were obtained from Centers for Diseases and Control (CDC, Atalanta, USA).

#### Larval habitat surveys and rearing of adult mosquito for testing the insecticide:

Thousands of *Anopheles* Larvae and pupae were collected from productive artificial larval habitats in Dire Dawa, Eastern Ethiopia site using a standard dipper of 350ml pouring to larval jar. *Anopheles species* pupae were separately counted and immersed a 250ml beaker in Cage. Emerged Adults about ten to fifteen were randomly screened for Microscopic morphological identifications before conducting the test.

We used the updated WHO 2023[28] guideline to perform WHO Bottle bioassay at which for each insecticide, six 250ml Wheaton bottles were washed, dried and of which four were coated with insecticide and the remaining coated with Acetone/Mero-Acetone as control bottles.

### Bottle coating and Insecticide preparations

For each insecticide testing four 250ml Wheaton bottles treated with the specific doses of insecticide and two 250ml Wheaton bottles used as controls and coated with acetone for CFP and PPF and mero-acetone for BRF and CLT as per the revised WHO protocol [28]. The detailed working solution and stock solution preparation are annexed (S1).

### Exposing mosquitoes to insecticides/testing

Twenty-five adult female *An. stephensi*, aged 2–5 days in separate six paper cups were prepared and each batch of 25 were transferred to insecticide coated wheaten bottle, each bottle has label in the cover and on-side indicating date of coating, replicate number and insecticide type (name and concentration) and controls.

In the case of Pyriproxyfen, equal number of mosquitoes were used for both test and control (100 each). Starved adult female were let blood fed before the test for 1 hour and coating was done then after they fed and the bottles were opened and covered with Aluminum foil to avoid any direct UV lights for two hours and then all the two hundred fed mosquitoes, in a batch of 25 per paper cup were exposed to respective coated replicates.

After 60 minutes of exposure, any knocked down mosquito were recorded and re-transferred to it’s own labeled paper cups for holding periods of 24hrs, 48 hrs and 72hrs [29].

But for the PPF holding, follow up holding period extended to seven days [26]. For the purpose of ovary dissection, checking oviposition and staging using Christopher’s method under high power magnification (40x-100x) under dissecting microscope[30], individual mosquito were transferred to each paper cup containing rinsed filter paper and cotton inside the bottom and followed until eggs get laid to seven days.

Exposure temperature and relative humidity were measured using thermo Hygro-meter and after 60’ minute recording the recording were re-set for 24 hr holding period. Recording for PPF continued to 72 hours. The testing temperature ranged from 25 to 29°C (temperature were monitored using heater) and the relative humidity at 65 to 85% were kept using humidifier. The mosquitoes were provided 10% sugar solution with cotton after completing the exposure period.

### Data analysis

Data were entered to SPSS V21 and analysed, the Turkey’s HSD multiple comparisons and Pearson’s Chi-square test results were used to compare the relationship between mosquito mortality on *An. stephensi* post exposure against the novel insecticides. The mean percentage oviposition and oviposition inhibition were compared to evaluate the sterilizing effect of PPF among exposed and non-exposed Adult *An.stephensi*.

## Results

Total of 650 female *An.stephensi* adults reared from larval/pupal collections were used to conduct the insecticide susceptibility test (N: 400 exposed and N: 250 control).

Complete mosquito mortality was observed in Brofilanilide and clothianidin after 24hours of exposure. The sixity minute 60 min knock down data showed 85%, 76%,14% and zero *An. stephensi* were knocked in response to exposure to BRF,CLT,CFP and PPF respectively.

**Figure 3:**
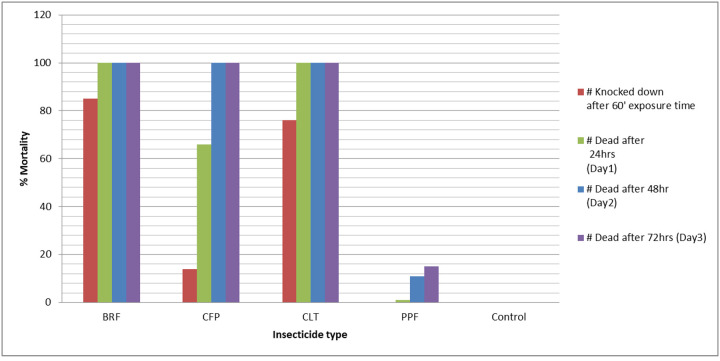
Result of Insecticide susceptibility status of *An. stephensi* against novel insecticides, Diredawa,60minute KD,mortality at 24hr, 48hr and 72 hr.

Figure 3, depicts the results of the susceptibility test at which *An. stephensi* were highly susceptible to novel insecticides of clothianidin, chlorfenapyr and Broflanilide but not for Pyriproxyfen.

All the three insecticides indicated > 60% mortality after 24hrs of exposure, 100% at subsequent 48 and 72hrs follow up periods and indicates how fast those insecticides can conquer to kill or make tired of *An. stephensi*.

Although these novel insecticides indicated higher mortality than PPF and control, there is a significance difference in each insecticide to knock or kill at each observation time.

BRF caused the highest KD(N:100,85%) and mortality(N:100,100%), followed by CLT which indicated KD(N:100,76%) and mortality(N:100,100%) at 24hours post exposure. But CFP showed KD(N:100,14%), mortality after 24 hrs (N:100,66%) and mortality at 48hrs(N:100,100%). No KD, only 1% mortality after 24hrs 11% mortality at 48hr and 15%mortality at 72 hrs were observed in *An.stephensi* exposed to PPF.No mortality was observed in controls (Table 1).

The ANOVA and multiple comparisons of Tukey’s honestly significance difference data analysis at post hock indicated that there was a significance difference (p < 0.05) in mean percent mortality in all insecticides after 24hrs of exposure. Except PPF, at which mortality was as low mortality as control and no significance difference (Mean difference = 0, p = 1).

As indicated in Table 1, PPF neither acted as a quick killer of exposed mosquitoes nor long acting. Rather causing inhibition of egg laying which was determined after five to seven days of follow up and up on ovary dissection.

PPF showed more of affecting the oviposition of *An. stephensi* up on ovary dissection at 7 days after exposure and causing only 15% of mortality after 72hours (Table 2).

Ovary dissection and staging of ovary development were assessed using Cristopher’s staging [30] key at high power 40x dissecting microscope and observations indicated all the 100 of the controls dissected at 7 day indicated stage IV ovaries well developed and eggs were observed.

No inhibitory impact of acetone were observed in controls but equal number of *An.stephensi* exposed to PPF indicated 100% inhibition and the ovary dissection indicated no production of eggs, or developed ovules (Table 2).

The sterilizing effect of PPF on *Anstephensiwere* observed on exposed groups and Chi-square test and Fisher’s exact test indicated that there is a strong correlation with sterility (oviposition inhibition) in exposed groups (*P value* < 0.05) than the non-exposed controls.

## Discussion

The possible efficacy of BRF, CFP, CLT and PPF were evaluated against wild *An. stephensi* reared from larval/pupal collections from artificial urban larval habitats in Diredawa, Ethiopia. This study demonstrated that resistant *An. stephensi* were susceptible (Mortality > 98%) to those novel insecticides and besides this study assessed the sterilizing effect of PPF against spreading *An.stephensi* (> 98% oviposition inhibition) using the WHO bottle bioassay.

Although a recent study conducted in Ethiopia and Afghanistan, indicated the level and mechanisms of insecticide resistance in *An. stephensi* against pyrethroids, organophosphates and carbamates using WHO Tube test is evident [8, 13, 31], in contrast, our study indicated the susceptibility to new novel insecticides using WHO Bottle bioassay.

In agreement with the WHO Multi-country study on insecticide susceptibility of *An. stephensi*, our study indicated 100% Mean mortality against CFP, CLT and comparable oviposition inhibition against PPF [29]. Similar studies conducted in Benin also showed comparable results of sterilizing effect although done in *An gambiae s.l*[22].

In Ethiopia, the susceptibility of *An arabiensis* against the novel insecticides were reported but not conducted against *An. stephensi* [32]. Similarly, a recent study conducted in Ethiopia on an update on the bionomics and susceptibility of *An.stephensi* indicated that *Anstephensiwere* highly resistant to pyrethroids, Carbamates and organophosphates but it indicated they were susceptible to PBO post pyrethroid exposure [8, 13], nevertheless they used WHO tube test method over WHO bioassay test and they also recommended to study the susceptibility status using CFP.

In contrast to our result on the sterilizing effect of PPF on *An. stephensi*, a research conducted by Aiku [33] indicated that there was no any sterilizing effect of 2% PPF exposed to *An. stephensi* compared with control groups but other study evaluated and presented that sterilizing adult malaria vectors using PPF could custom part of a malaria control strategy as there is the lack of reported resistance to PPF in mosquitoes and its unique mode of action [34]. Similarly, in Burkina Faso, a study done on *An. gambiae* kisimu showed that PPF caused irreversible complete inhibition of fertility (100%) [35].

Therefore, the report of this study provided the susceptibility status of *An. stephensiagainst* the novel insecticides using the revised WHO Bottle bioassay test.

### Limitation

The susceptibility study was done using larvae/pupae of *An. stephensi* populations collected and raised form artificial habitats from Urban site, wish to include periurban and rural sites to find if there exist a population variation across the clusters.

## Conclusion

This study indicated that *An. stephensi were* fully susceptible to the three novel insecticides: Clorfenapyr, clothianidin and Broflainilide. In addition, Pyriproxyfen have caused 100% oviposition inhibition in exposed groups than controls. Therefore, it is recommended to use these novel insecticides to control adult *An. stephensi in* Ethiopia.

## Figures and Tables

**Figure 2:**
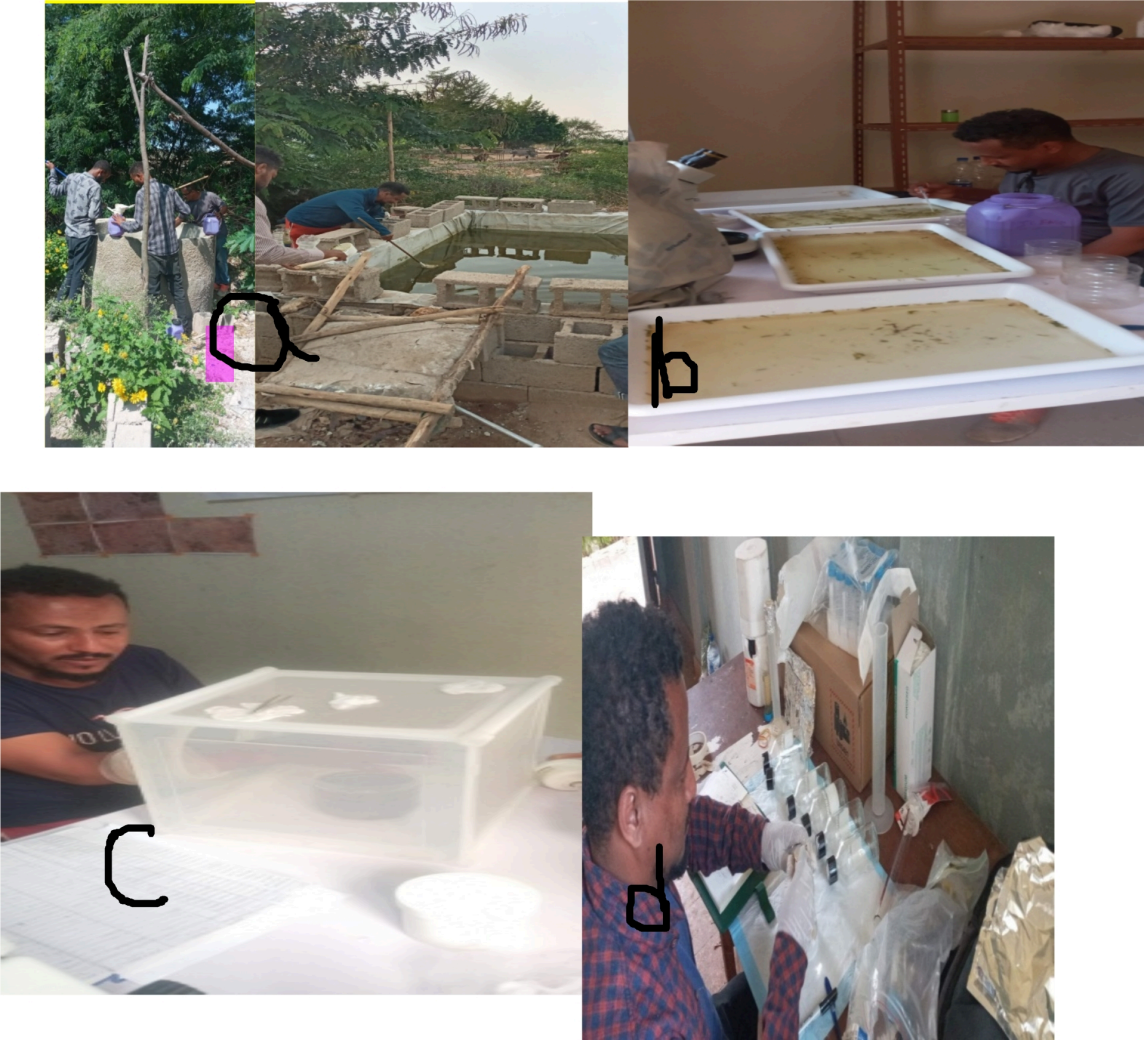
a)Larval/pupal collection in an artificical habitat,b)Pupal counting ,C)Arm feeding for PPF Exposure d) Bottle Coating and testing

**Table 1:** Susceptibility test result of Anopheles stephensiin diagnostic assay, Diredawa, 2024

Type of assay	Insecticide Class	Insecticide	Concentration	Percentage mortalityat 24hr (number tested)	Percentage Mortality (number tested) at 48hr	Percentage Mortality (number tested) at 72hr	WHO Criteria
Diagnostic assay	Meta-di-amide	Broflanilide	9ug	100(100)	100(100)	100(100)	Susceptible
	Pyrrol	Cholrfenapyr	100ug	66(100)	100(100)	100(100)	Susceptible
	Neo-nicotiniod	Clothianidin	10ug	100(100)	100(100)	100(100)	Susceptible
	Insect growth regulator (IGR)	Pyriproxyfen*	100ug	1 (100)	11(100)	15(100)	Susceptible*

* PPF is an insect growth regulator and according to WHO PPF guideline if Oviposition inhibition is ≥ 98%, indicates susceptible.

**Table 2:** Oviposition and Oviposition inhibition of Wild Anstephensifrom Dire Dawa after exposure to 100ug PPF and Acetone using WHO bottle bioassay, 2024

Strain	Test	Chambers tested at 96hr	# of *An.stephensi* dissected and positives for eggs at 120hr N (%)	#of *An.stephensi* dissected and positives for eggs at 144hr N (%)	#of *An.stephensi* dissected and positives for eggs at 168hr N (%)	Oviposition inhibition N (%)
*An.stepehensi* wild	PPF	85	25(0)	25(0)	30(0)	85(100%)
	Acetone	100	25(100)	35(100)	40(100)	100(0)

## Data Availability

The datasets used and/or analyzed during the current study are available from the corresponding author on reasonable request.

## Abbreviations

AI: Activie ingredient
An: Anopheles
BRF: Broflanilide
CFP: Chlorfenapyr
CLT: chlothianidin
hr: Hour
IRS: Indoor residual spraying
ITNs: Insecticide treated nets
KD: Knock down
OP: organophosphate
PBO: piperynol butoxide
PF: Plasmodium falciparum
PPF: pyriproxyfen
PY: pyrethroid
PV: Plasmodium vivax
s.l: senso lato
WHO: World Health Organization

## References

[1] Gaugler R. Medical entomology for students. Oxford University Press; 2016.

[2] Organization WH. World malaria report 2023. World Health Organization; 2023.

[3] Wesolowski R, Plasmodium knowlesi as a threat to global public health. Korean J Parasitol. 2015;53(5):575. 26537037 10.3347/kjp.2015.53.5.575 PMC4635839

[4] Taylor R, Invasive Anopheles stephensi in Africa: insights from Asia. Trends in Parasitology; 2024. 10.1016/j.pt.2024.06.008 39054167

[5] Hamlet A, The potential impact of Anopheles stephensi establishment on the transmission of Plasmodium falciparum in Ethiopia and prospective control measures. BMC Med. 2022;20(1):135. 35440085 10.1186/s12916-022-02324-1 PMC9020030

[6] Carter TE, First detection of Anopheles stephensi Liston, 1901 (Diptera: culicidae) in Ethiopia using molecular and morphological approaches. Acta Trop. 2018;188:180–6. 30189199 10.1016/j.actatropica.2018.09.001

[7] Balkew M, Geographical distribution of Anopheles stephensi in eastern Ethiopia. Parasites vectors. 2020;13:1–8. 31959237 10.1186/s13071-020-3904-y PMC6971998

[8] Balkew M, An update on the distribution, bionomics, and insecticide susceptibility of Anopheles stephensi in Ethiopia, 2018–2020. Malar J. 2021;20(1):263. 34107943 10.1186/s12936-021-03801-3 PMC8189708

[9] Ashine T, Spatiotemporal distribution and bionomics of Anopheles stephensi in different eco-epidemiological settings in Ethiopia. Parasites Vectors. 2024;17(1):166. 38556881 10.1186/s13071-024-06243-3 PMC10983662

[10] Massebo F The expansion of an invasive malaria vector: Anopheles stephensi detection in Arba Minch town in the southern Rift Valley of Ethiopia. 2024. 10.1007/s00436-024-08356-1 PMC11436467 39331165

[11] Hawaria D, First report of Anopheles stephensi from southern Ethiopia. Malar J. 2023;22(1):373. 38066610 10.1186/s12936-023-04813-x PMC10704791

[12] Yared S, Insecticide resistance in Anopheles stephensi in Somali Region, eastern Ethiopia. Malar J. 2020;19:1–7. 32398055 10.1186/s12936-020-03252-2 PMC7216317

[13] Samake JN Insecticide resistance and population structure of the invasive malaria vector, Anopheles stephensi, from Fiq. Ethiopia bioRxiv, 2024: p. 2024.08. 21.609030. 10.1038/s41598-024-78072-4 PMC11554808 39528579

[14] Acford-Palmer H, Identification of two insecticide resistance markers in Ethiopian Anopheles stephensi mosquitoes using a multiplex amplicon sequencing assay. Sci Rep. 2023;13(1):5612. 37019918 10.1038/s41598-023-32336-7 PMC10076309

[15] Tadesse FG Anopheles stephensi mosquitoes as vectors of Plasmodium vivax and falciparum, Horn of Africa, 2019. Emerging infectious diseases, 2021. 27(2): p. 603. 10.3201/eid2702.200019 PMC7853561 33496217

[16] Wilson ML, Urban malaria: understanding its epidemiology, ecology, and transmission across seven diverse ICEMR network sites. Am J Trop Med Hyg. 2015;93(3 Suppl):110. 26259941 10.4269/ajtmh.14-0834 PMC4574269

[17] Raghavendra K, Chlorfenapyr: a new insecticide with novel mode of action can control pyrethroid resistant malaria vectors. Malar J. 2011;10:1–7. 21266037 10.1186/1475-2875-10-16 PMC3039634

[18] Verma V, Chlorfenapyr: Irritant effect compared to other insecticides and its intrinsic toxicity in multiple-insecticide-susceptible and-resistant: Anopheles stephensi:(Diptera: Culicidae). J Vector Borne Dis. 2015;52(1):99–103. 25815874

[19] Uragayala S, Adulticidal & larvicidal efficacy of three neonicotinoids against insecticide susceptible & resistant mosquito strains. Indian J Med Res. 2015;142(Suppl 1):S64–70. 26905244 10.4103/0971-5916.176624 PMC4795349

[20] Nakao T, Banba S. Broflanilide: A meta-diamide insecticide with a novel mode of action. Bioorg Med Chem. 2016;24(3):372–7. 26361738 10.1016/j.bmc.2015.08.008

[21] Portwood NM, Multi-centre discriminating concentration determination of broflanilide and potential for cross-resistance to other public health insecticides in Anopheles vector populations. Sci Rep. 2022;12(1):22359. 36572746 10.1038/s41598-022-26990-6 PMC9792579

[22] Abai MR, Malaria control activities in Iran and novel evaluation of pyriproxyfen as an insect growth regulator (IGR) against malaria vectors in a malarious area. J Entomol Zool Stud. 2019;7(4):27–31.

[23] Mishra A. Effect of pyriproxyfen against Dipterans as a growth regulator. Int J Multidisciplinary Res, 2022. 10(3).

[24] Myers A, Identifying suitable methods for evaluating the sterilizing effects of pyriproxyfen on adult malaria vectors: a comparison of the oviposition and ovary dissection methods. Malar J. 2024;23(1):164. 38789998 10.1186/s12936-024-04983-2 PMC11127354

[25] Soto A, Ovary dissection is a sensitive measure of sterility in Anopheles gambiae exposed to the insect growth regulator pyriproxyfen. Insects. 2023;14(6):552. 37367368 10.3390/insects14060552 PMC10299475

[26] WHO. Standard operating procedure for evaluating the sterilizing properties of pyriproxyfen in adult female mosquitoes in WHO bottle bioassay. 2022.

[27] Zoungbédji DM, Assessing the susceptibility and efficacy of traditional neurotoxic (pyrethroid) and new-generation insecticides (chlorfenapyr, clothianidin, and pyriproxyfen), on wild pyrethroid-resistant populations of Anopheles gambiae from southern Benin. Malar J. 2023;22(1):245. 37626366 10.1186/s12936-023-04664-6 PMC10463682

[28] Organization WH. Standard operating procedure for testing insecticide susceptibility of adult mosquitoes in WHO bottle bioassays. 2022.

[29] Corbel V, A new WHO bottle bioassay method to assess the susceptibility of mosquito vectors to public health insecticides: results from a WHO-coordinated multi-centre study. Volume 16. Parasites & vectors; 2023. p. 21. 1. 36670470 10.1186/s13071-022-05554-7 PMC9863080

[30] WHO. Manual on practical entomology in Malaria. Part II. Methods and techniques. 1975.

[31] Safi NHZ, Status of insecticide resistance and its biochemical and molecular mechanisms in Anopheles stephensi (Diptera: Culicidae) from Afghanistan. Malar J. 2019;18:1–12. 31349836 10.1186/s12936-019-2884-x PMC6660931

[32] Dagg K, Evaluation of toxicity of clothianidin (neonicotinoid) and chlorfenapyr (pyrrole) insecticides and cross-resistance to other public health insecticides in Anopheles arabiensis from Ethiopia. Malar J. 2019;18:1–11. 30795768 10.1186/s12936-019-2685-2 PMC6387473

[33] Aiku A, Yates A, Rowland M. Laboratory evaluation of pyriproxifen treated bednets on mosquito fertility and fecundity. A preminary study. West Afr J Med. 2006;25(1):22–6. 16722354 10.4314/wajm.v25i1.28240

[34] Harris C, Sterilising effects of pyriproxyfen on Anopheles arabiensis and its potential use in malaria control. Parasites vectors. 2013;6:1–8. 23683439 10.1186/1756-3305-6-144 PMC3669104

[35] Koama B, The sterilizing effect of pyriproxyfen on the malaria vector Anopheles gambiae: physiological impact on ovaries development. Malar J. 2015;14:1–8. 25880844 10.1186/s12936-015-0609-3 PMC4355148

